# Partial factorial trials: comparing methods for statistical analysis and economic evaluation

**DOI:** 10.1186/s13063-018-2818-x

**Published:** 2018-08-16

**Authors:** Helen A. Dakin, Alastair M. Gray, Graeme S. MacLennan, Richard W. Morris, David W. Murray

**Affiliations:** 1 Health Economics Research Centre, Nuffield Department of Population Health, Old Road Campus, Headington, Oxford, OX3 7LF UK; 2 Centre for Healthcare Randomised Trials, University of Aberdeen, Aberdeen, UK; 3 School of Social and Community Medicine, University of Bristol, Bristol, UK; 4 Nuffield Department of Orthopaedics, Rheumatology and Musculoskeletal Sciences, University of Oxford, Oxford, UK

**Keywords:** Randomised controlled trial, Factorial design, Cost-utility analysis, Bayesian bootstrap, Partial factorial trial

## Abstract

**Background:**

Partial factorial trials compare two or more pairs of treatments on overlapping patient groups, randomising some (but not all) patients to more than one comparison. The aims of this research were to compare different methods for conducting and analysing economic evaluations on partial factorial trials and assess the implications of considering factors simultaneously rather than drawing independent conclusions about each comparison.

**Methods:**

We estimated total costs and quality-adjusted life years (QALYs) within 10 years of surgery for 2252 patients in the Knee Arthroplasty Trial who were randomised to one or more comparisons of different surgical types. We compared three analytical methods: an “at-the-margins” analysis including all patients randomised to each comparison (assuming no interaction); an “inside-the-table” analysis that included interactions but focused on those patients randomised to two comparisons; and a Bayesian vetted bootstrap, which used results from patients randomised to one comparison as priors when estimating outcomes for patients randomised to two comparisons. Outcomes comprised incremental costs, QALYs and net benefits.

**Results:**

Qualitative interactions were observed for costs, QALYs and net benefits. Bayesian bootstrapping generally produced smaller standard errors than inside-the-table analysis and gave conclusions that were consistent with at-the-margins analysis, while allowing for these interactions. By contrast, inside-the-table gave different conclusions about which intervention had the highest net benefits compared with other analyses.

**Conclusions:**

All analyses of partial factorial trials should explore interactions and assess whether results are sensitive to assumptions about interactions, either as a primary analysis or as a sensitivity analysis. For partial factorial trials closely mirroring routine clinical practice, at-the-margins analysis may provide a reasonable estimate of average costs and benefits for the whole trial population, even in the presence of interactions. However, such conclusions will be misleading if there are large interactions or if the proportion of patients allocated to different treatments differs markedly from what occurs in clinical practice. The Bayesian bootstrap provides an alternative to at-the-margins analysis for analysing clinical or economic endpoints from partial factorial trials, which allows for interactions while making use of the whole sample. The same techniques could be applied to analyses of clinical endpoints.

**Trial registration:**

ISRCTN, ISRCTN45837371. Registered on 25 April 2003.

**Electronic supplementary material:**

The online version of this article (10.1186/s13063-018-2818-x) contains supplementary material, which is available to authorized users.

## Background

Full factorial trials randomise all patients to any combination of two or more treatments: e.g. A, B, both or neither. Partial factorial trials^1^ also evaluate multiple treatments simultaneously on the same patient group, but randomise only a subset of patients to two or more factors, while other patients are randomised to just one factor or to a different combination of factors [1–3]. For example, a full factorial trial may randomise all patients to drug A or its placebo and simultaneously to drug B or its placebo, while a partial factorial trial may randomise some patients to drug A or its placebo, randomise some to drug B or its placebo and randomise other patients simultaneously to A or its placebo *and* to B or its placebo (Table 1). High-profile examples of this design include the Women’s Health Initiative [2] and the United Kingdom Prospective Diabetes Study (UKPDS) [4].

**Table 1:** Schematics of hypothetical full and partial factorial designs

Full factorial trial (*n* = 400)						
	Placebo of A		Drug A		Total	
Placebo of B	100 pts. (*00*)		100 pts. (*a0*)		200 pts	
Drug B	100 pts. (*0b*)		100 pts. (*ab*)		200 pts	
Total	200 pts		200 pts			
Partial factorial trial (*n* = 400)						
		Randomised in comparison A		Not randomised in comparison A		Total
		Placebo of A	Drug A	Did not have A	Received A	
Randomised in comparison B	Placebo of B	40 pts. (*00*)	40 pts. (*a0*)	30 pts. (*−0*)	40 pts. (*+ 0*)	150 pts
	Drug B	40 pts. (*0b*)	40 pts. (*ab*)	28 pts. (*−b*)	42 pts. (*+b*)	150 pts
Not randomised in comparison B	Did not have B	10 pts. (*0–*)	11 pts. (*a–*)			
	Received B	40 pts. (*0+*)	39 pts. (*a+*)			
Total		130 pts	130 pts			

By comparing multiple treatment factors on overlapping populations, partial factorial trials can address multiple questions in the same study, reducing the fixed costs of each research question and the overall sample size compared with conducting several non-overlapping trials. Since some patients are randomised to two or more factors in a factorial manner, partial factorial trials can also investigate interactions: i.e. whether the effect of A differs depending on whether B is also given. Partial factorial designs are often used when economic, geographic or clinical constraints restrict the comparisons to which patients can be randomised [1]. In particular, this design facilitates flexible recruitment strategies, such as letting patients [2] or clinicians [5] choose which comparisons to be randomised into, or recruiting only patients from certain countries [6] or those with specific comorbidities, clinical characteristics or laboratory findings to a second (or even third) comparison [2, 7].

While there is extensive research and established guidelines on full factorial trials, to date only one review article has discussed partial factorial trials [1]. Partial factorial trials raise several additional issues over and above those introduced by full factorial designs, and the appropriate analytical methods are less clear and under-researched.

As well as evaluating the “main effects” of each factor, it is also informative to estimate the magnitude of interactions and evaluate the impact of interactions on the conclusions, particularly for economic endpoints. Increasing numbers of economic evaluations are conducted alongside randomised trials [8, 9]. Recent work has suggested that interactions between treatments are particularly likely to arise for economic endpoints [10, 11]. This work also demonstrated the importance of making a joint decision between all combinations of treatments (e.g. between A, B, neither or both) allowing for interactions, rather than making separate decisions on treatments for the same patient group that assume no interaction [11]. We therefore evaluated methods using an economic evaluation based on a partial factorial trial, although similar issues also apply to analyses of clinical endpoints.

We propose four methods that could be used to estimate main effects in partial factorial trials, with or without allowance for interactions:

1. *At-the-margins analysis*. Analysing each factor separately (e.g. evaluating drug A in Table 1 by comparing outcomes for the 130 patients in cells *a0*, *ab, a*– and *a*+ with those for the 130 patients in cells *00*, *0b, 0*– and *0*+) may give a good indication of population average effects for partial factorial trials, although this approach is prone to bias when there are interactions [12–14]. Several reviews on full factorial trials argue that at-the-margins estimates of the main effect of factor A may be informative even in the presence of interactions if the ratio of B to not-B in the trial reflects the ratio in the setting of interest (e.g. routine clinical practice) [12, 15, 16]. While this is unlikely for many full factorial trials, partial factorial trials enable the distribution of patients between B and not-B to be governed by routine clinical practice for those patients not randomised to this comparison (although conversely it is also possible that clinicians’ use of B will be affected by whether patients are also randomised to receive A). As a result, at-the-margins analysis may give a good estimate of average incremental effects across the whole population for a partial factorial trial, even if there is an interaction.
2. *Inside-the-table analysis*. This focuses on the subset of patients randomised to > 1 comparison and analyses cells *00*, *a0*, *0b* and *ab* (Table 1) “inside-the-table” like a full factorial trial. This analysis ensures unbiased estimation of interactions, but it excludes many patients (60% [240/400] in the case of Table 1). Consequently, interactions can only be evaluated on a small sample, and the power to detect main effects is reduced. Furthermore, the patients randomised to > 1 comparison may not be representative of the whole trial population, potentially reducing generalisability.
3. *Bayesian bootstrap*. This previously-described technique [17, 18] could be used to update the inside-the-table analysis on patients randomised to > 1 comparison (cells *00*, *a0*, *0b* and *ab* in Table 1) to take account of the additional information provided by patients randomised to only one comparison (cells *0*–, *0*+, *a*–*, a*+, *− 0,* + *0*, *−b* and + *b*). This approach uses the entire sample “as-randomised”. Bayesian bootstrapping has recently been applied to take account of external evidence from other trials [18], but it has not previously been applied to partial factorial studies.
4. *Subgroup analysis*. This analyses the entire trial population “as-treated” and subdivides all patients into ≥ 4 groups based on the combination of treatments that they actually received. For example, in Table 1 we might pool cells *ab* and *a*+. Inside-the-table analysis can therefore be done by comparing outcomes for the four combinations of received treatment. However, this approach analyses patients according to the treatment that they actually received (rather than their randomised allocation) and is therefore prone to the selection bias associated with per protocol analysis [19]. Furthermore, since the patients in cells *0*–, *0*+, *a*–*, a*+, *− 0,* + *0*, *−b* and + *b* are not randomly assigned to the second factor, any observed effect of this second factor or any observed interactions between the two factors could be caused by confounding rather than causal effects. For example, if we find that patients in cell *0*+ have worse outcomes than those in cell *0*–, this could be due to patient characteristics that both affected outcomes and the probability of receiving treatment B, rather than a causative effect of B. As such, subgroup analysis of partial factorial trials carries many of the same hazards and biases as analyses of observational studies or subgroup analysis of two- or three-arm trials [20, 21]. By contrast, full factorial randomisation with intention-to-treat (ITT) analysis and concealment of allocation avoids selection bias and ensures that the only systematic difference between randomised groups is the allocated treatment.

This study aims to explore the implications of partial factorial design on the methods and results of economic evaluation, illustrate the methods that can be applied and compare how the results and conclusions of an applied example differ between at-the-margins analysis, inside-the-table analysis on patients randomised to > 1 comparison and the Bayesian bootstrap. The subgroup approach is presented in Additional file 1 due to the bias inherent in this approach. Additional file 1 also summarises the assumptions underpinning each analysis.

## Methods

### Case study

The Knee Arthroplasty Trial (KAT, International Standard Randomised Trial No. ISRCTN45837371; see Additional file 2) is a pragmatic partial factorial randomised trial evaluating three^2^ aspects of knee prosthesis design [3, 5, 22]:

1. *Bearing*. Using a mobile bearing versus a fixed bearing
2. *Backing*. Using a metal-backed tibial component versus one made of solid polyethylene
3. *Patella*. Resurfacing the patella (i.e. replacing part of the knee cap with plastic) versus no resurfacing

**Fig. 1:**
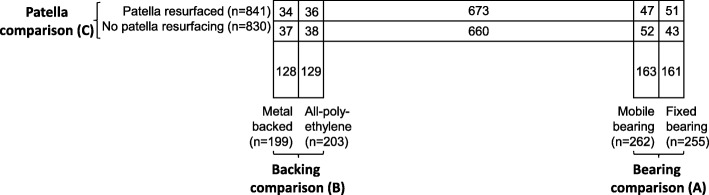
Design of KAT (*n* = 2252, excluding patients who died/withdrew before surgery). *Numbers* and *rectangular areas* represent the number of patients randomised to each arm. Adapted from Table 1 in Murray et al. [3]

Patients about to undergo knee replacement were recruited and randomised to those comparisons for which the surgeon was in equipoise. The partial factorial design enabled patients to be randomised to the patella comparison as well as either the bearing or backing comparison (Fig. 1). This design and recruitment strategy was chosen to maximise recruitment of surgeons and patients and to acknowledge the marked variations between surgeons in the comparisons for which they are willing to accept randomisation. Given that there was no previous evidence on interactions and no clinical reason why one would be expected (at least for the primary endpoint, Oxford knee score), the primary clinical and economic analyses were conducted at-the-margins [3]. This follows standard practice for most factorial trials and would be orthodox for any simple treatment-versus-no-treatment comparison in any randomised trial, even where interactions with subgroups were plausible.

Cost-utility analysis, calculating the cost per quality-adjusted life year (QALY) gained, was conducted on results at a median of 10 years’ post-operation follow-up [3] (see Additional file 1). Net monetary benefit (NMB = QALYs ⋅ Rc ‐ cost) is reported at a £20,000/QALY ceiling ratio (Rc) to reflect the amount that the National Health Service (NHS) is typically willing/able to pay to gain one QALY [23]. Costs are presented in 2011/2012 pounds.

Multiple imputation [24] was used to impute missing data on utilities and resource use. To ensure a fair comparison, the same set of imputed values was used for all analyses. Imputation was conducted on the entire trial population rather than separately for each comparison and assumed no interactions to match the base case analysis (see Additional file 1).

Bootstrapping was conducted to allow for uncertainty. For Analyses 1, 2 and 4, we used 100 bootstraps on each of the 100 imputed datasets and based point estimates on the sample mean (with no bootstrapping or bias correction); results for the 100 imputed datasets were combined using Rubin’s rule [24] and used to estimate standard errors (SEs), *p* values and cost-effectiveness acceptability curves (CEACs). The bootstrapping methods used in Analysis 3 are described below.

With the exception of Analysis 3, all analyses were conducted in Stata version 12. All *p* values are two-sided.

### Analysis 1 (base case) methods: at-the-margins analysis

The base case analysis comprised at-the-margins analysis to ensure consistency with the primary clinical analysis and to take account of all participants as-randomised. Furthermore, at-the-margins estimates are particularly likely to reflect population average effects for KAT, since patients were only randomised to the comparisons for which surgeons were in equipoise, which implies that treatment allocation would have been approximately 50:50 regardless of whether patients were randomised. For this analysis, bootstrapping was conducted separately for each of the three comparisons, including only those patients randomised to that comparison.

### Analysis 2 methods: inside-the-table analysis

Analysis 2 considered the subset of patients randomised to > 1 comparison on an ITT basis like a small full factorial trial. In this analysis, bootstrapping was conducted twice: once on the 193 patients randomised to the bearing and patella comparisons, and once on the 145 patients randomised to the backing and patella comparisons. For each bootstrap, linear regression was used to predict the costs and QALYs accrued in each year of the trial:

1 $$ {\displaystyle \begin{array}{l} AnnualCost={\beta}_0+{\beta}_AA+{\beta}_{Patella} Patella+{\beta}_{Int} Patella\cdot A\\ {} AnnualQALYs={\beta}_0+{\beta}_AA+{\beta}_{Patella} Patella+{\beta}_{Int} Patella\cdot A+{\beta}_{BaselineUtility} BaselineUtility\end{array}} $$

where *A* indicates randomised allocation in the bearing or backing comparisons, and *Patella* indicates whether patients were randomised to patella resurfacing. The interaction between treatment allocations was calculated for total costs, total QALYs and NMB in each bootstrap replicate (Interaction = *μ*_ab_ − *μ*_*a*0_ − *μ*_0*b*_ + *μ*_00_, where *μ*_*x*_ indicates the mean outcome in arm *x*).

### Analysis 3 methods: Bayesian bootstrap

Bayesian bootstrapping involves weighting bootstrap samples based on a prior. This can be done using rejection sampling, where the weights determine the probability that a bootstrap sample is included in the analysis rather than being rejected [18, 25]. We used this “vetted bootstrap” technique to explore the interaction between interventions in a partial factorial trial while taking account of the entire sample, including the patients randomised to only one comparison (who were excluded from inside-the-table analysis).

We used the (posterior) evidence from the patients randomised to one comparison as a prior that was updated using the evidence from patients randomised to > 1 comparison. This was implemented by rejecting a proportion of the bootstraps on patients randomised to > 1 comparison that had at-the-margins incremental NMB (INB) estimates that were not consistent with the data on patients randomised to one comparison. For simplicity, rejection sampling was done using INB, which combines between-group differences in costs and QALYs into a single metric that reflects value for money. Costs, QALYs and NMB were then calculated for the set of bootstraps that passed rejection sampling based on their INB. The analysis was done in five steps (steps 1, 2a–c and 3).

Step 1: Estimate outcomes for patients randomised in only one comparison that are used as priors for rejection sampling in step 2b. Patients who were randomised in > 1 comparison were excluded from this analysis to ensure that the priors were independent of the data that were used to update them. We calculated the mean INB across each of the three sets of patients randomised to only one comparison ( *INB*_*Ae*_ = *NMB*_*A*_ − *NMB*_*notA*_ for those patients randomised only in comparison A, and similarly for *INB*_*Be*_ and *INB*_*Ce*_). We then conducted standard bootstrap analyses on the same three samples, drawing 100 bootstrap samples from each of the 100 imputed datasets. SEs around mean INB (*σ*_*Ae*_, *σ*_*Be*_ and *σ*_*Ce*_) were then calculated using Rubin’s rule [24].

Step 2a: Bootstrap samples of patients randomised to > 1 comparison. We conducted a standard bootstrap on the two sets of patients randomised to > 1 comparison, conducting > 300 bootstraps per imputed dataset on the patients randomised to the bearing and patella comparisons and 750 bootstraps per imputed dataset for the patients randomised to the backing and patella comparisons.^3^

Step 2b: Calculate weights for each bootstrap sample from step 2a. The weights determine the probability that this bootstrap sample would be included in the analysis. The weights *ω*(*INB*_*A*_, *INB*_*C*_)^∗^ comprise numbers between 0 and 1 that indicate how closely the INB for the current bootstrap of patients randomised to > 1 comparison (${INB}_{A^{\ast }}$ and ${INB}_{C^{\ast }}$) matches the evidence (*INB*_*Ae*_ and *INB*_*Ce*_) calculated on patients randomised to one comparison. We followed [18] in assuming a Gaussian likelihood:

2 $$ \omega {\left({INB}_A,{INB}_C\right)}^{\ast }=\exp \left(-\frac{{\left({INB}_{A^{\ast }}-{INB}_{Ae}\right)}^2}{2{\sigma}_{Ae}^2}-\frac{{\left({INB}_{C^{\ast }}-{INB}_{Ce}\right)}^2}{2{\sigma}_{Ce}^2}\right) $$

Weights were based on INB, rather than costs or QALYs, since allowing for the correlation between costs and QALYs would have complicated the estimation of weights. The calculation of weights takes advantage of the fact that INB for patients randomised only to comparison A (*INB*_*Ae*_) is independent of the INB for patients randomised only to C (*INB*_*Ce*_). The base case results were based on rejection sampling using a £20,000/QALY ceiling ratio, although this ceiling ratio was varied to generate CEACs.

For a partial factorial trial, it is debatable whether weights should be based on *main effects* (calculated at-the-margins, e.g. as the average of *a0* and *ab* in Table 1, minus the average of *00* and *0b*) or *simple effects* (calculated inside-the-table, e.g. as *a0* minus *00*) for the patients randomised in > 1 comparison. In this case, we felt that the INB for patients randomised only to mobile versus fixed bearing was more comparable to the at-the-margins estimate of the INB for mobile versus fixed bearing for the patients randomised to > 1 comparison, since both include patients with a mixture of patella resurfacing and no resurfacing (and likewise for the other comparisons). However, the most appropriate approach should be decided on a case-by-case basis, since in other situations (e.g. where one of the treatments is not used at all in the patients randomised to only one comparison), it might be more appropriate to use the simple effect.

Step 2c: Use weights to determine which bootstraps from step 2a are included in the analysis. The weights were compared against numbers randomly drawn from a uniform distribution, and those bootstraps with random numbers greater than the weight were excluded from the analysis. We considered each bootstrap independently; as a result, the analysis included more bootstraps from some imputed datasets than others.

Step 3: Analyse the bootstraps passing the vetting. We calculated the mean NMB, cost and QALYs for each intervention by averaging across the bootstraps passing rejection sampling. We developed a modified version of Rubin’s rule (Eq. 3), which calculates standard errors ($\sqrt{\mathrm{v}\widehat{\mathrm{a}}\mathrm{r}\left(\widehat{\theta}\right)}$) as the weighted average of the variance ($\mathrm{v}\widehat{\mathrm{a}}\mathrm{r}\left({\widehat{\theta}}_m\right)$) and the deviation from the overall mean (${\left({\widehat{\theta}}_m-\widehat{\theta}\right)}^2$) for each of the M imputed datasets, giving equal weight to each bootstrap that passed the rejection sampling:

3 $$ \mathrm{v}\widehat{\mathrm{a}}\mathrm{r}\left(\widehat{\theta}\right)=\frac{1}{M\overline{N}}\sum \limits_{m=1}^M\mathrm{v}\widehat{\mathrm{a}}\mathrm{r}\left({\widehat{\theta}}_m\right){N}_m+\left(1+\frac{1}{M}\right)\left(\frac{1}{M\overline{N}-\overline{N}}\right)\sum \limits_{m=1}^M{\left({\widehat{\theta}}_m-\widehat{\theta}\right)}^2{N}_m $$

where *N*_*m*_ indicates the number of imputed datasets passing rejection sampling in imputation *m*, while $\overline{N}$ equals the average number of bootstraps included across all *M* imputed datasets. The derivation is given in Additional file 1.

Bayesian *p* values were based on the proportion of included bootstraps with positive or negative values for the parameter of interest. CEACs were based on the proportion of bootstraps in which each treatment combination had the highest NMB. Bootstrapping was conducted in Stata version 14, while subsequent analyses were conducted in Microsoft Excel 2010. Additional file 3 shows the methods used to estimate weights and vet bootstraps in Excel. We conducted steps 2a–c sequentially, conducting all bootstraps before determining which were included in the analysis, although these steps may be done for each bootstrap in turn, discarding results for bootstraps that did not pass the vetting.

## Results

### Analysis 1 (base case) results: at-the-margins analysis

Analysis 1 evaluated each of the three comparisons separately on all patients randomised to that comparison, treating each comparison as an independent decision. Comparing outcomes for all patients randomised to patella resurfacing against all those randomised to no resurfacing suggested that patella resurfacing dominated no resurfacing, generating an additional 0.19 QALYs and saving an average of £104 per patient treated with a 96% chance of being cost-effective^4^ (Table 2, Additional file 1). On average, mobile bearings were cost-effective compared with fixed bearings, although QALY gains were very small and there was substantial uncertainty around this finding. Analysis 1 also suggested that we can be 91% confident that metal-backed components are cost-effective compared with non-metal-backed ones, with an incremental cost-effectiveness ratio (ICER) of just £35 per QALY gained. If we were to make separate decisions treating the different aspects of knee component design as independent options, we might therefore recommend patella resurfacing, metal backing and (more hesitantly) mobile bearing.

**Table 2:** Results of at-the-margins analysis for all three comparisons

	Comparison A: mobile bearing (*n* = 262) versus fixed bearing (*n* = 255)	Comparison B: metal-backed (*n* = 199) versus non-metal-backed (*n* = 203)	Comparison C: Patella resurfacing (*n* = 841) versus no resurfacing (*n* = 830)
Treatment group			
Cost	£8998 (£310)	£8235 (£272)	£8785 (£161)
QALYs	5.007 (0.143)	5.219 (0.151)	5.297 (0.076)
NMB^a^	£91,145 (£2968)	£96,145 (£3112)	£97,158 (£1551)
Control group			
Cost	£8913 (£405)	£8225 (£344)	£8889 (£211)
QALYs	4.956 (0.141)	4.926 (0.152)	5.110 (0.080)
NMB^a^	£90,209 (£2938)	£90,290 (£3144)	£93,308 (£1662)
Difference			
Cost	£85 (£508)	£10 (£440)	–£104 (£269)
QALYs	0.051 (0.196)	0.293 (0.210)	0.187 (0.108)
NMB^a^	£936 (£4087)	£5854 (£4343)	£3849 (£2235)
ICER (per QALY gained)	£1666	£35	Dominant
Probability cost-effective^a^	0.59	0.91	0.96
Probability cost-saving	0.42	0.47	0.64

Numbers in brackets are SEs

^a^At a £20,000/QALY ceiling ratio

### Analysis 2 results: inside-the-table analysis

Analysis 2 aimed to inform joint decisions between combinations of treatment strategies by analysing the subset of patients randomised to > 1 comparison inside-the-table as a full factorial trial. The analysis of mobile bearings and patella resurfacing included 193 patients: 37% of those randomised in the bearing comparison. The subset of patients randomised to both comparisons tended to have higher costs than the average patient in the bearing comparison, and all SEs were at least twice as large as those in Analysis 1 due to the substantially smaller sample size (Tables 2 and 3).

**Table 3:** Results of comparison A (mobile versus fixed bearing) for Analyses 2 and 3

	Analysis 2: inside-the-table	Analysis 3: Bayesian bootstrap
With patella resurfacing		
Mobile bearing (*N* = 47)^a^		
Cost	£9068 (£466)	£9112 (£468)
QALYs	5.559 (0.264)	5.471 (0.227)
NMB^b^	£102,110 (£5372)	£100,301 (£4612)
Fixed bearing (*N* = 51)^a^		
Cost	£9169 (£1165)	£9382 (£1167)
QALYs	4.959 (0.289)	4.891 (0.240)
NMB^b^	£90,015 (£6256)	£88,443 (£5169)
No patella resurfacing		
Mobile bearing (*N* = 52)^a^		
Cost	£11,100 (£1147)	£10,930 (£1065)
QALYs	4.732 (0.311)	4.820 (0.251)
NMB^b^	£83,533 (£6755)	£85,475 (£5385)
Fixed bearing (*N* = 43)^a^		
Cost	£8481 (£464)	£8416 (£460)
QALYs	5.029 (0.294)	5.135 (0.231)
NMB^b^	£92,104 (£6014)	£94,290 (£4703)

Numbers in brackets are SEs

^a^In Analysis 3, the bootstraps were weighted using weights calculated using a sample of 324 patients randomised only in the bearing comparison (163 to mobile bearing and 161 to fixed bearing) and 1333 patients randomised only in the patella comparison (673 to patella resurfacing and 660 to no resurfacing)

^b^At a £20,000/QALY ceiling ratio

Large, but not statistically significant, interactions were observed for costs, QALYs and NMB (Table 4). All three interactions were qualitative, with the incremental costs, QALYs and NMB for mobile bearings changing sign depending on whether patients were allocated to patella resurfacing or no resurfacing (Table 3). In particular, patients randomised to mobile bearings with no patella resurfacing accrued substantially higher costs and substantially fewer QALYs than the other three groups. Mobile bearings therefore dominated fixed bearings in patients who were also randomised to patella resurfacing, but were dominated in patients randomised to no resurfacing. However, making a joint decision about mobile bearings and patella resurfacing based on this analysis and adopting the treatment with the highest expected NMB nonetheless suggested that mobile bearings with patella resurfacing should be recommended — the same conclusion obtained from at-the-margins analysis.

**Table 4:** Magnitude of interactions in Analyses 2 and 3

	Analysis 2: inside-the-table analysis	Analysis 3: Bayesian bootstrapping
Comparison A: mobile versus fixed bearing		
Interaction for cost (SE)	–£2720 (£1751), *p* = 0.12	–£2784 (£1790), *p* = 0.09
Interaction for QALYs (SE)	0.90 (0.51), *p* = 0.08	0.89 (0.51), *p* = 0.08
Interaction for NMB^a^ (SE)	£20,667 (£10,820), *p* = 0.06	£20,672 (£11,029), *p* = 0.06
Comparison B: metal-backed versus all-polyethylene		
Interaction for cost (SE)	£506 (£907), *p* = 0.58	£475 (£890), *p* = 0.58
Interaction for QALYs (SE)	1.06 (0.54), *p* = 0.05	1.12 (0.53), *p* = 0.03
Interaction for NMB^a^ (SE)	£20,788 (£11,094), *p* = 0.06	£21,940 (£10,954), *p* = 0.05

^a^At a £20,000/QALY ceiling ratio

**Fig. 2:**
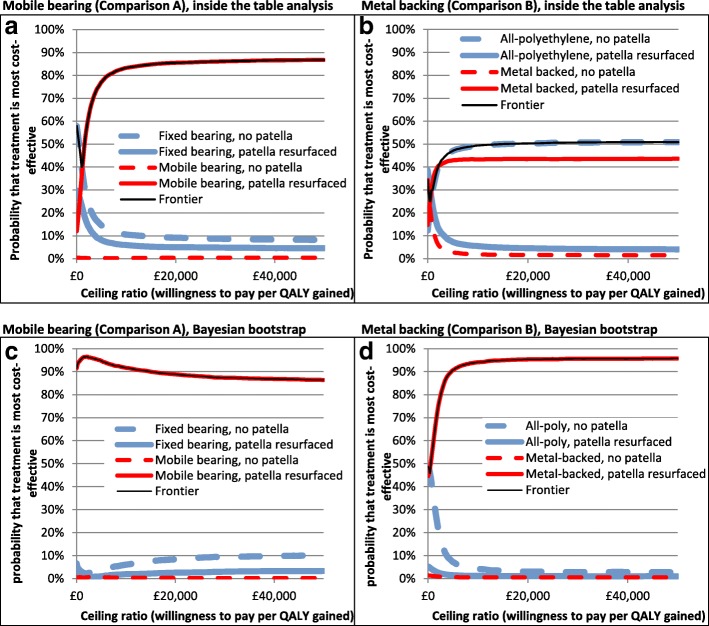
CEACs for multiple comparisons allowing for interactions

However, despite the smaller sample size, inside-the-table analysis suggested that there is substantially less uncertainty around this decision compared with at-the-margins analysis because the expected NMB for mobile bearings with patella resurfacing is much higher than that of the alternatives. Mobile bearing plus patella resurfacing had an 86% chance of being cost-effective in this analysis (Fig. 2a), whereas in Analysis 1, the probability of mobile bearings being cost-effective was only 59%.

Inside-the-table analysis on the 145 patients randomised to the metal backing and patella comparisons also highlighted a statistically significant interaction for QALYs and non-significant qualitative interactions for costs and NMB (Table 4).

Both costs and QALYs were substantially higher in the group randomised to no metal backing and no patella resurfacing and the group randomised to metal backing and patella resurfacing than in the other two groups (Table 5). Furthermore, this analysis suggested that the treatment with the highest NMB is all-polyethylene with no patella resurfacing, with the opposite combination (metal backing with patella resurfacing) coming a close second. However, the subset of patients randomised in both the backing and patella comparisons tended to accrue lower costs and higher QALYs than those randomised to just the backing comparison, suggesting that this patient population may not be typical.

**Table 5:** Results of comparison B (metal-backed versus all-polyethylene) for Analyses 2 and 3

	Analysis 2: inside-the-table	Analysis 3: Bayesian bootstrap
With patella resurfacing		
Metal backing (*N* = 34)^a^		
Cost	£8036 (£411)	£7925 (£391)
QALYs	5.518 (0.337)	5.820 (0.248)
NMB^b^	£102,327 (£6870)	£108,467 (£5041)
All-polyethylene (*N* = 36)^a^		
Cost	£7833 (£567)	£7849 (£550)
QALYs	5.046 (0.330)	4.989 (0.262)
NMB^b^	£93,087 (£6837)	£91,940 (£5416)
No patella resurfacing		
Metal backing (*N* = 37)^a^		
Cost	£7782 (£384)	£7749 (£357)
QALYs	4.976 (0.311)	5.117 (0.258)
NMB^b^	£91,745 (£6348)	£94,586 (£5255)
All-polyethylene (*N* = 38)^a^		
Cost	£8085 (£409)	£8148 (£409)
QALYs	5.569 (0.248)	5.407 (0.220)
NMB^b^	£103,293 (£5031)	£99,999 (£4451)

Numbers in brackets are SEs

^a^In Analysis 3, the bootstraps were weighted using weights calculated using a sample of 257 patients randomised only in comparison B (128 to metal backing and 129 to all-polyethylene) and 1333 patients randomised only in comparison C (673 to patella resurfacing and 660 to no resurfacing)

^b^At a £20,000/QALY ceiling ratio

There was also substantial uncertainty around this finding (Fig. 2b), with a 43% chance that metal backing with patella resurfacing has the highest NMB and a 50% chance that all-polyethylene with no patella resurfacing is best.

### Analysis 3 results: Bayesian bootstrap

Bayesian bootstrapping produced estimates of mean costs, QALYs and NMB that were broadly similar to those of Analysis 2, although SEs were lower in Analysis 3 for QALYs and NMB in all groups and for costs in most groups (Tables 3 and 5). The magnitude of interactions and their standard errors were similar to those for inside-the-table analysis, and both analyses found all interactions to be qualitative (Table 4).

Like Analyses 1 and 2, Bayesian bootstrapping found mobile bearing with patella resurfacing to be the most effective and best value for money intervention in the bearing comparison, having the highest expected NMB and highest mean QALYs. However, Bayesian bootstrapping found metal backing with patella resurfacing to be the most cost-effective option in the backing comparison. This matches the results of at-the-margins analysis, but contradicts the unexpected finding of inside-the-table analysis (which found all-polyethylene with no resurfacing to have a slightly higher NMB than metal backing with patella resurfacing).

CEACs demonstrated that Bayesian bootstrapping produced results with substantially less uncertainty around the most appropriate treatment option than inside-the-table analysis, because the INB for the most cost-effective treatment compared with the next best alternative was larger with a smaller standard error. The probability that mobile bearings with patella resurfacing are cost-effective was above 86% at all ceiling ratios (Fig. 2c), indicating that there is substantially less uncertainty around this conclusion in this analysis than was suggested by Analyses 1 or 2. The probability that metal backing with patella resurfacing was cost-effective was also above 94% at all ceiling ratios above £10,000/QALY gained (Fig. 2d), in stark contrast to inside-the-table analysis, where there was substantial uncertainty about whether the best treatment was metal backing with patella resurfacing or all-polyethylene with no resurfacing (Fig. 2b).

## Discussion

In this paper, we have developed and evaluated three methods for estimating interactions within partial factorial trials and compared them with at-the-margins analysis. Each analysis provided different estimates of incremental costs, QALYs and NMB. Conclusions for the backing comparison also varied between analyses: inside-the-table analysis suggested that all-polyethylene with no patella resurfacing was best, whereas at-the-margins analysis, Bayesian bootstrapping and the subgroup analysis (described in Additional file 1) found metal backing with patella resurfacing to be best.

Analysing the subset of patients randomised to > 1 comparison, inside-the-table (Analysis 2) provides an unbiased estimate of interactions, since patients are analysed in the groups to which they were randomised, ensuring that there is no systematic difference between groups other than the treatment they received. However, by restricting analysis to those in > 1 comparison, this analysis included only 15% of the KAT trial population, greatly increasing SEs and substantially reducing the power to detect differences between treatment groups. Furthermore, the subset of patients included in > 1 comparison may not be typical of the overall trial population. In this case, patients who were in both the backing and patella comparisons tended to accrue lower costs and higher QALYs than those randomised to one comparison, with the opposite trends among patients in the bearing and patella comparisons. Consequently, the interactions observed in these patient subgroups may not generalise to the wider population, and the unexpected finding that all-polyethylene and no patella resurfacing has the highest NMB could be spurious or due to chance. However, inside-the-table analysis may have greater power and greater generalisability in trials that randomise a greater proportion of patients to > 1 comparison than was the case in KAT.

At-the-margins analysis (Analysis 1) may provide a useful estimate of average costs and benefits for the population of interest even when interactions exist. This is particularly relevant to KAT, since decisions about those aspects of implant design that were not randomly assigned reflected routine clinical practice, and patients were randomised only to those comparisons about which surgeons were in equipoise. Indeed, the proportion of patients having patella resurfacing in KAT (50%) was similar to that in routine clinical practice (39% [26]). Furthermore, at-the-margins analysis is simple to conduct and explain, and it maximises statistical power by analysing the entire trial population. However, by ignoring interactions, at-the-margins analysis gives biased estimates of the effect of each treatment in isolation [13]: in this case, the bias in NMB estimates (equal to half the size of the interaction term [13]) was around £10,000, which is up to ten times larger than the main effects. Furthermore, if the interaction between metal backing and patella resurfacing observed in inside-the-table analysis were genuine (i.e. if all-polyethylene with no patella resurfacing truly has a higher NMB than metal backing with patella resurfacing), ignoring this interaction and basing decisions on at-the-margins analysis would fail to maximise health gains from the budget.

Bayesian bootstrapping (Analysis 3) overcomes the drawbacks of the other three techniques, using all of the data, analysing patients as-randomised and avoiding bias by taking account of interactions. This approach assumes that the evidence collected in patients randomised to one comparison is applicable to patients randomised to > 1 comparison, although in principle it would be possible to down-weight the evidence from the former group. In particular, evidence from patients randomised only in comparison A (for whom treatment B was not randomly allocated) is used to vet the bootstraps before we estimate outcomes for comparison B, although outcomes are always analysed as-randomised. This is similar to using external evidence from an A-versus-no-A trial alongside a factorial trial on A and B. It may therefore be prudent to present a sensitivity analysis using inside-the-table or at-the-margins analysis alongside Bayesian bootstrapping — especially for regulatory submissions. Bayesian methods also enable interactions to be down-weighted using sceptical priors [27, 28], providing a compromise between including or excluding interaction terms. Rejection sampling is a useful way to implement the Bayesian bootstrap for economic evaluations, since it facilitates estimation of CEACs. However, importance sampling [18, 25], whereby a weighted average is calculated across the bootstraps rather than rejecting a proportion of bootstraps, may be more convenient and computationally faster when analysing clinical endpoints from partial factorial trials.

Although the subgroup analysis presented in Additional file 1 (Analysis 4) considered all patients, it required the patella comparison to be analysed as-treated, not as-randomised, which introduced selection bias. Consequently, the markedly smaller interactions observed in this analysis may be due to, or confounded by, patient characteristics (e.g. age, physical activity or severity of bone damage) that affect whether surgeons undertake patella resurfacing as well as the costs and QALYs accrued over the time horizon.^5^ This analysis therefore does not inform causal inferences about the main effect of patella resurfacing or interactions between factors and is not an appropriate way to evaluate or control for interactions in partial factorial trials.

This study suggests that there is evidence that patella resurfacing may affect the incremental costs, QALYs and cost-effectiveness of mobile bearings and metal backing. Interactions were generally qualitative (changing the direction of incremental effects). Although interactions were not expected a priori, it is plausible that since patella resurfacing, mobile bearings and metal backing can influence knee kinematics, interactions could occur that might affect patients’ quality of life and/or their risk of readmission. However, the exact form of the interactions is difficult to explain clinically: for example, it is unclear why all-polyethylene with patella resurfacing should produce fewer QALYs than either metal backing with patella resurfacing or all-polyethylene without resurfacing. KAT was not designed or powered to estimate interactions; generally interactions need to be twice as large as the predicted main effect to be detected with the same statistical power [14]. Furthermore, the observed interactions could be due to chance, since only three of the 18 interaction terms estimated were statistically significant at the 0.05 level. Nonetheless, such interactions may warrant further investigation. The present study is the first methodological work conducted on partial factorial trials and applies methods to a particular dataset, but further work simulating a variety of plausible scenarios would add to our understanding of the use and analysis of partial factorial designs and help to evaluate the analytical methods in datasets where the “true” interaction is known.

## Conclusions

This study demonstrates that partial factorial trials can be used to evaluate interactions. Such trials are more informative and cheaper than conducting two separate trials on non-overlapping populations. However, compared with full factorials, a partial factorial design reduces the size and generalisability of the sample available for evaluating interactions. Nonetheless, a partial factorial design may substantially increase recruitment in situations where not all patients can be randomised to > 1 comparison, thereby increasing the size and generalisability of the sample available for at-the-margins analysis with little/no impact on the number randomised to > 1 comparison. Nonetheless, given the difficulties evaluating interactions, partial factorial designs may be best reserved for situations where interactions are expected to be negligible.

The choice of analysis may depend on the decision-maker’s research question and the impact of interactions. At-the-margins analysis may provide a useful estimate of average treatment effects for pragmatic partial factorial trials in which the proportion of patients receiving each treatment is likely to be similar to that in routine clinical practice. Nonetheless, at-the-margins analysis is prone to bias and potentially misleading conclusions whenever interactions exist [10, 12–14], so the impact of interactions should always be evaluated in sensitivity analysis. Furthermore, unlike the other methods, at-the-margins only estimates main effects and does not provide any information on interactions. For economic evaluation, it is generally more appropriate to make a joint decision about which combination of treatments provides the best value for money, taking account of any interactions [10, 11]. Such decisions are facilitated by inside-the-table analysis and Bayesian bootstrapping, whereas at-the-margins analysis implicitly assumes no interaction and only informs separate decisions on each factor.

Bayesian bootstrapping may be the most appropriate way to evaluate interactions in partial factorial trials, although conducting inside-the-table analysis on patients randomised to > 1 comparison may provide a simple method for conducting sensitivity analysis, particularly for studies where most patients are randomised to > 1 comparison. Evaluating interactions on the total sample by subgrouping patients by the treatment they receive is inappropriate, as it breaks randomisation and is prone to bias.

The principles and methods developed here for economic endpoints could also be applied to analyses of any outcome measure to assess the magnitude of interactions and the extent to which the results are sensitive to interactions.

## Additional files

Additional file 1: Additional information on assumptions, methods, acceptability curves, methods and results of the inside-the-table subgroup analysis (DOCX 190 kb)

Additional file 2: CONSORT checklist and flowcharts for the KAT trial. (DOCX 42 kb)

Additional file 3: Spreadsheet demonstrating the methods used to vet bootstraps. Gives the formulae used to estimate weights, vet the bootstraps and analyse results, using a small number of hypothetical bootstraps that would normally be generated in another software package (e.g. Stata). (XLSX 422 kb)

## Abbreviations

CEAC: Cost-effectiveness acceptability curve
INB: Incremental net (monetary) benefit
IPW: Inverse probability weighting
ITT: Intention-to-treat
KAT: Knee Arthroplasty Trial
NMB: Net monetary benefit
QALY: Quality-adjusted life year
SE: Standard error

## Footnotes

1 We follow the definition used by [1–3], although the term “partial factorial trials” is also used to describe “incomplete factorial trials” in which all experimental units are randomised to a subset of the possible combinations of factors [12, 29].

2 A fourth factor was also evaluated [3], comparing total versus unicompartmental knee replacement, but it is not discussed here since it did not overlap with other comparisons and stopped early due to difficulties in recruiting participants.

3 This number of bootstraps was chosen for pragmatic reasons and ensured that after rejection sampling there were at least 6000 bootstraps included in the analysis at a £20,000/QALY ceiling ratio, although the exact number of bootstraps included in the calculation of CEACs varied with ceiling ratio.

4 Notably, this analysis provides a real-life example of the situation identified hypothetically previously [30, 31], in which NMB differs significantly (*p* = 0.96 on a one-tailed test) between treatments, despite non-significant differences in both costs and effects.

5 In principle, we could reduce bias by adjusting for observed confounders (e.g. using propensity scores or genetic matching [32, 33]), although the subgroup approach would remain vulnerable to bias from unobserved confounders.
