# Supplementary material for: Partial factorial trials: comparing methods for statistical analysis and economic evaluation
Source: Trials. 2018 Aug 16;19:442. doi: 10.1186/s13063-018-2818-x (PMC6097309; doi:10.1186/s13063-018-2818-x)
Supplement: Supplementary file 1 — Additional information on assumptions, methods, acceptability curves, methods and results of the inside-the-table subgroup analysis (DOCX 190 kb) [file 13063_2018_2818_MOESM1_ESM.docx]

**Additional file 1: Additional information on assumptions, methods, acceptability curves and methods and results of the inside-the-table subgroup analysis**

**Assumptions used in each analysis**

**Table S1.** Assumptions used in each analysis

|  | **1: At-the-margins** | **2: Inside-the-table** | **3: Bayesian bootstrapping** | **4: Subgroup analysis** |
| --- | --- | --- | --- | --- |
| Parameters estimated | Main effects | Main effects, interactions, outcomes for each treatment combination | Main effects, interactions, outcomes for each treatment combination | Main effects, interactions, outcomes for each treatment combination |
| Patients included for the bearing comparison | All patients randomised in the bearing comparison | Those patients randomised in both the bearing comparison and the patella comparison | All patients randomised in the bearing comparison and those randomised only in patella comparison | All patients randomised in the bearing comparison |
| Assumptions | - No interaction between factors: i.e. additive effects - The proportion of patients in comparison A of the trial who also received B reflects the proportion having B in the setting of interest: differences between groups therefore reflect population average effects even in presence of interactions | - The patients randomised to >1 comparison are representative of the population of interest | - Evidence on patients randomised to only 1 comparison also applies to those in 2 comparisons (although this could be relaxed by adjusting the weights) - Mean INB in the groups of patients randomised to only 1 comparison has a Gaussian sampling distribution (although weights could be based on any continuous distribution) | - The effect of each factor is causal: a very strong assumption in this case where only 1 factor is randomly assigned and patients are analysed as-treated, rather than as-randomised - No confounding factors influence protocol deviations or which treatment is given to patients who were randomised in only 1 comparison |

**Methods of the cost-utility analysis**

We conducted a within-trial cost-utility analysis from the perspective of the UK National Health Service (NHS); full details have been reported previously [1]. We prospectively collected data on NHS resource use related to the study knee for each trial participant, including the hospital stay for the primary operation and any related readmissions, theatre costs for the primary operation and any subsequent surgery, knee replacement components, blood transfusions, CT/ultrasound scans, and general practice, physiotherapy and outpatient consultations related to the study knee. Resources were valued using unit costs reported previously [1] using an index year of 2011/12; where necessary prices were adjusted for inflation [2, 3].

Quality-adjusted life-years (QALYs) estimated from EQ-5D utilities using the UK N3 time-trade-off tariff [4] were used as a measure of health outcomes to capture the magnitude and duration of changes in quality of life. EQ-5D utility was measured before surgery, three months after surgery, 12 months after surgery and annually thereafter. QALYs were calculated for each patient as the area under the curve, interpolating linearly between EQ-5D measurements.

The analysis took a 10 year time horizon; costs and QALYs incurred after Year 1 were discounted at 3.5% per year [5]. We used inverse probability weighting (IPW) [6] to allow for administrative censoring. All four analyses presented in this paper also used linear regression to adjust QALYs for imbalance in baseline utility [7].

Following the analyses described in the Methods, bootstrapping results were used to estimate cost-effectiveness acceptability curves showing the proportion of bootstraps (across all imputed datasets) in which treatment had highest NMB or generated cost savings. Further details of methods for the cost-utility analysis are given on pages 8-19 of the monograph by Murray et al [1].

**Multiple imputation in partial factorial trials**

Partial factorial designs raise particular challenges for multiple imputation. Firstly, imputation could be conducted once on the entire trial population, or separately for each comparison. We used the former approach in this study to maximise the amount of data available for imputation and thereby get more precise imputations and conditional imputation models. This also facilitates Analyses 2-4 and ensures consistency between comparisons and between analyses. The alternative strategy of conducting imputation separately for each of the three comparisons would have greatly reduced the sample size for imputation models (and therefore the precision of imputed values) and produced two different sets of imputed values for patients randomised to two comparisons.

Secondly, the best way to define treatment variables in the imputation model is unclear. To avoid bias, the variables, functional forms and interaction terms in any imputation model should match those used in the analysis model [8]. For full factorial trials, variables for all potential analysis models can be captured by including one variable for each factor and one for each interaction term. However, for partial factorial trials, having one dummy per factor is not feasible: patients can, for example, be randomised to mobile bearing, randomised to fixed bearing or not randomised in the bearing comparison (in which case they may receive either mobile or fixed bearings). We therefore included six treatment dummies in the imputation model (RandtoPatella, RandtoNoPatella, RandtoMobile, RandtoFixed, RandtoMetal, RandtoPoly), each equal to 1 if the patient was randomised to that treatment and 0 if they were randomised to the alternative or not randomised in that comparison:

$$if(Randto\text{Patella}=1,orRandto\text{NoPatella}=1) ,then:y=\beta_{0}+\beta_{\text{Patella}}Randto\text{Patella}$$

$=\beta_{\text{PatellaNoCons}}Randto\text{Patella}+\beta_{\text{NoPatellaNoCons}}Randto\text{NoPatella}$ (A1)

where y is costs or QALYs. This coding is equivalent to an at-the-margins model and does not allow for interactions between factors; it may therefore cause inside-the-table analysis and Bayesian bootstrapping to underestimate the magnitude of interactions. In principle, interaction terms (RandtoPatella*RandtoMobile and RandtoPatella*RandtoMetal) could have been added into the model, although they may have hindered convergence of the imputation model, since <50 patients would have a 1 in each interaction term.

Full details of the imputation model used in this analysis have been reported previously [1]. In addition to assuming no interaction between factors, we assumed that data were missing at random.

**Derivation of modified Rubin’s rule for Bayesian bootstrap with rejection sampling**

The standard Rubin’s rule calculates point estimates by averaging across M imputed datasets and calculates the variance (i.e. standard error squared) as:

 (A2)

When we conduct a Bayesian bootstrap with rejection sampling, the number of bootstraps included in the analysis varies between imputed datasets. Applying the standard formulae for Rubin’s rule would give equal weight to each imputed dataset, rather than being based on the weights used in rejection sampling. Instead, we derived a modified version of Rubin’s rule that gives equal weight to each bootstrap that passed the rejection sampling.

Point estimates were calculated simply by averaging across all bootstraps passing rejection sampling. We modified the formula for calculating standard errors as the weighted average of the variance () and deviation from the overall mean () for each of the M imputed datasets, using weights equal to the number of imputed datasets passing rejection sampling in imputation m ($N_{m}$):

 (A3)

where $\bar{N}$ equals the average number of bootstraps included across all M imputed datasets.

**Methods for Analysis 4: Secondary analysis of interactions as subgroup analysis**

In addition to analysing KAT at-the-margins (Analysis 1), inside-the-table on those patients randomised to >1 comparison (Analysis 2) and using Bayesian bootstrapping (Analysis 3), a sensitivity analysis was conducted using the subgroup approach described in the Introduction.

Analysis 4 comprised a subgroup analysis of all patients randomised to either the bearing comparison or the backing comparison, evaluating the impact of mobile bearings and metal backing in patients with and without randomisation in the patella comparison (C). Whereas Analyses 1-3 were conducted on a strict ITT basis, for Analysis 4, it was necessary to subdivide patients based on actual patella treatment received, rather than randomised allocation, since patella resurfacing was not randomly allocated in 63% (581/919) of patients in the bearing or backing comparisons. Patella treatment received was identified from the components used in the procedure; seven patients (0.76%) missing all component codes were excluded from this analysis as it was impossible to assess whether or not the patella was resurfaced.

The OLS models used to predict costs or QALYs accrued in each year were the same as those for Analysis 2, except that *Patella* indicates whether or not the patella was resurfaced, rather than randomised allocation to patella resurfacing:

$\begin{matrix} AnnualCost=\beta_{0}+\beta_{A}A+\beta_{Patella}Patella+\beta_{Int}Patella\cdot A \\ AnnualQALYs=\beta_{0}+\beta_{A}A+\beta_{Patella}Patella+\beta_{Int}Patella\cdot A+\beta_{BaselineUtility}BaselineUtility \end{matrix}$ (A4)

Where *A* indicates randomised allocation in the bearing or backing comparisons

The bearing and backing comparisons were analysed separately, with regression analyses being repeated on 100 bootstrap replicates of each of the 100 imputed datasets. The impact of interactions was evaluated in the same way as for Analysis 2.

**Additional results of Analysis 1: At-the-margins analysis**

**Figure A1.** CEACs for independent decisions based on at-the-margins analysis: including all patients randomised in each comparison.


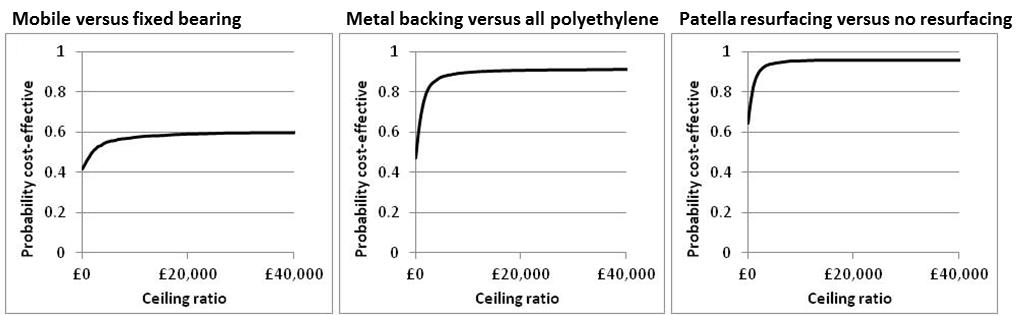
Reproduced from Figures 22, 38 and 54 in Murray et al 2014 [1].

**Results of Analysis 4: Subgroup analysis of interactions**

Analysis 4 evaluated interactions in a larger patient group by comparing the incremental effect of metal backing or mobile bearings in patients who received patella resurfacing with the incremental effect in patients who received no resurfacing. Since this analysis included all but seven patients randomised in the bearing comparison, the at-the-margins estimate of the effect of mobile vs. fixed bearings (averaged across patients with patella resurfacing and those without) in this sample was almost identical to that from Analysis 1 (Table A2). However, since 19 patients did not receive their allocated patella treatment and this analysis includes only 12% (193/1671) of patients randomised in the patella comparison, the costs and benefits for the patella comparison differ substantially from the base case analysis. Most SEs were slightly larger than those observed in Analysis 1, but smaller than those in Analyses 2 or 3.

**Table S2.** Subgroup analysis of interactions: including all patients randomised to the bearing or backing comparisons

|  | | **Mobile bearing** | **Fixed bearing** | **Metal backing** | **All-polyethylene** |
| --- | --- | --- | --- | --- | --- |
| Patella resurfaced | No. pts | 121 | 125 | 99 | 98 |
|  | Cost | £8,413 (£234) | £9,289 (£763) | £8,843 (£426) | £8,134 (£548) |
|  | QALYs | 5.131 (0.187) | 5.141 (0.179) | 5.290 (0.170) | 4.873 (0.202) |
|  | NMB* | £94,202 (£3,815) | £93,526 (£3,857) | £96,949 (£3,527) | £89,319 (£4,247) |
| No patella resurfacing | No. pts | 140 | 128 | 98 | 103 |
|  | Cost | £9,522 (£545) | £8,555 (£349) | £7,620 (£321) | £8,329 (£436) |
|  | QALYs | 4.872 (0.182) | 4.768 (0.178) | 5.179 (0.202) | 5.025 (0.186) |
|  | NMB* | £87,918 (£3,882) | £86,810 (£3,651) | £95,969 (£4,132) | £92,164 (£3,826) |

* At a £20,000/QALY ceiling ratio.

Qualitative interactions between bearings and patella were observed for costs (-£1,842 [SE: £1,024]; p=0.72) and QALYs (-0.11 [SE: 0.31]; p=0.07), in addition to quantitative interactions for NMB (-£433 [SE: £6,680]; p=0.95). However all interaction terms were substantially smaller than those estimated in Analyses 2 or 3 and had smaller SEs. This analysis therefore suggests that the bias within at-the-margins estimates is substantially smaller than is suggested by Analyses 2 or 3 (-£216, vs. £10,334). The interactions meant that mobile bearings were less costly and less effective than fixed bearings in patients who had patella resurfacing, but more costly and more effective in those without resurfacing. However, mobile bearings with patella resurfacing nonetheless had highest expected NMB at a £20,000/QALY ceiling ratio. Since this treatment had lower expected NMB than in Analysis 2, the uncertainty about which treatment was best was substantially greater, despite the larger sample size and smaller SEs: there was a 52% chance that mobile bearings with patella resurfacing was the best treatment (Figure A2A; cf. >80% in Analyses 2 and 3).

**Figure S2.** CEACs for multiple comparisons based on subgroup analysis of interactions.


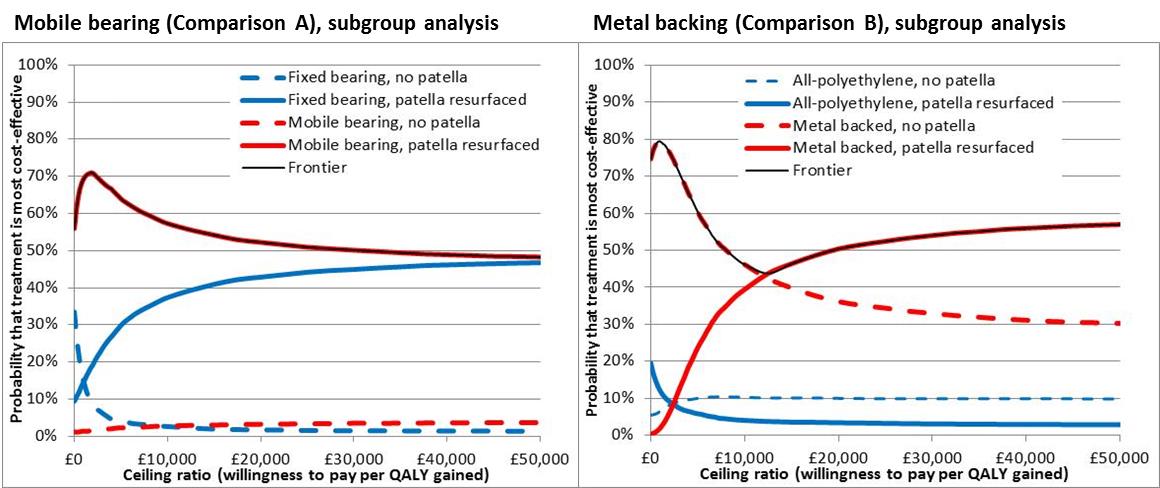


For the backing comparison, SEs estimated in Analysis 4 (Table A2) were substantially larger than those observed in Analysis 1, but generally smaller than those from Analyses 2 and 3. While the incremental costs and outcomes for metal backing vs. no metal backing matched Analysis 1, very different results were obtained for the patella comparison: in the subset of patients randomised in the backing comparison, those patients who received a patella accrued slightly fewer QALYs on average than those who did not undergo resurfacing.

There were qualitative interactions for costs (£1,419 [SE: £878]; p=0.11), QALYs (0.26 [SE: 0.32]; p=0.41) and NMB (£3,824 [SE: £6,698]; p=0.57), although these were generally smaller than those in Analyses 2 and 3 and not statistically significant, despite the substantially larger sample size. This analysis suggests that the bias within at-the-margins NMB estimates could be £1,912 (£3,824/2). The qualitative interaction for NMB meant that patella resurfacing was cost-effective in patients having metal backing, but not in those with all-polyethylene components, although metal backing was cost-effective regardless of patella resurfacing.

Metal backing with patella resurfacing had highest expected NMB; this conclusion matches the conclusion that was drawn from Analyses 1 and 3, but differs from the conclusions of Analysis 2 (which suggested that all-polyethylene with no patella resurfacing was best). However, there was also substantial uncertainty around the best treatment, which was largely driven by uncertainty about patella resurfacing: the probability that metal backing with patella resurfacing had highest NMB was just 50%, with a 36% probability that the treatment with second highest expected NMB (metal backing without patella resurfacing) was best (Figure A2B).

However, this analysis analysed the patella comparison as-treated, not as-randomised, which introduces substantial selection bias. As result, any observed interactions may be due to patient characteristics (e.g. age, physical activity or severity of bone damage) that affect the chance of surgeons choosing to undertake patella resurfacing as well as the costs and QALYs accrued over the time horizon. More fundamentally, we also cannot draw unbiased conclusions about the causative relationship between patella resurfacing and costs or QALYs because this analysis is per-protocol and in most patients the decision to resurface (or not) was based on patient characteristics and surgeon preference, rather than random allocation. This bias and the large interactions mean that Analysis 4 finds patella resurfacing to be (on average) dominated by no patella resurfacing in those patients randomised in the backing comparison, whereas Analyses 1-3 find patella resurfacing to be dominant. The main effect for the patella comparison is prone to similar selection bias as observational studies, which means that this analysis cannot usefully inform joint decisions about patella resurfacing and tibial design. The impact of this bias could be even greater for partial factorial designs in other clinical areas, such as those where only patients with high blood pressure are randomised to receive antihypertensive treatment or placebo.

**References**

1. Murray DW, MacLennan GS, Breeman S, Dakin HA, Johnston L, Campbell MK et al. A randomised controlled trial of the clinical effectiveness and cost-effectiveness of different knee prostheses: the Knee Arthroplasty Trial (KAT). Health Technology Assessment. 2014;18. doi:10.3310/hta18190.

2. Department of Health. Financial matters - May 2012 - Appendix 1 - HSCI March 2012 - Health service cost index. 2012. <http://www.info.doh.gov.uk/doh/finman.nsf/af3d43e36a4c8f8500256722005b77f8/7c011d898a440d7680257a21004b66ce?OpenDocument>. Accessed 8th August 2012.

3. Department of Health. Financial matters - September 2012 - Appendix 1 - HSCI July 2012 - Health service cost index. 2012. <http://www.info.doh.gov.uk/doh/finman.nsf/af3d43e36a4c8f8500256722005b77f8/023c449a382e219c80257a7d0041879d?OpenDocument>. Accessed 8th August 2012.

4. Dolan P. Modeling valuations for EuroQol health states. Med Care. 1997;35:1095-108.

5. National Institute for Health and Care Excellence. Guide to the methods of technology appraisal 2013. 2013. <http://www.nice.org.uk/media/D45/1E/GuideToMethodsTechnologyAppraisal2013.pdf>. Accessed 17th May 2013.

6. Gray A, Clarke P, Wolstenholme J, Wordsworth S. Chapter 7: Analysing costs. Applied Methods of Cost-Effectiveness Analysis in Health Care Handbooks in Health Economic Evaluation Series. Oxford: Oxford University Press; 2011.

7. Manca A, Hawkins N, Sculpher MJ. Estimating mean QALYs in trial-based cost-effectiveness analysis: the importance of controlling for baseline utility. Health Econ. 2005;14:487-96. doi:10.1002/hec.944.

8. White IR, Royston P, Wood AM. Multiple imputation using chained equations: Issues and guidance for practice. Stat Med. 2011;30:377-99. doi:10.1002/sim.4067.
