# Supplementary material for: Partial factorial trials: comparing methods for statistical analysis and economic evaluation
Source: Trials. 2018 Aug 16;19:442. doi: 10.1186/s13063-018-2818-x (PMC6097309; doi:10.1186/s13063-018-2818-x)
Supplement: Supplementary file 2 — CONSORT checklist and flowcharts for the KAT trial. (DOCX 42 kb) [file 13063_2018_2818_MOESM2_ESM.docx]

**Additional file 2: CONSORT checklist and flowcharts**

Table S3. CONSORT checklist [1] indicating where details of the KAT randomised trial are given in Murray et al 2014 [2].

| Section/Topic | Item No | Checklist item | Reported on page No |
| --- | --- | --- | --- |
| Title and abstract | | | |
|  | 1a | Identification as a randomised trial in the title | i |
|  | 1b | Structured summary of trial design, methods, results, and conclusions (for specific guidance see CONSORT for abstracts) | vii-viii, xxv-xxvii |
| Introduction | | | |
| Background and objectives | 2a | Scientific background and explanation of rationale | 1-2 |
|  | 2b | Specific objectives or hypotheses | 1 |
| Methods | | | |
| Trial design | 3a | Description of trial design (such as parallel, factorial) including allocation ratio | 3 |
|  | 3b | Important changes to methods after trial commencement (such as eligibility criteria), with reasons | 3 |
| Participants | 4a | Eligibility criteria for participants | 4 |
|  | 4b | Settings and locations where the data were collected | 21 |
| Interventions | 5 | The interventions for each group with sufficient details to allow replication, including how and when they were actually administered | 4-5 |
| Outcomes | 6a | Completely defined pre-specified primary and secondary outcome measures, including how and when they were assessed | 5-6 |
|  | 6b | Any changes to trial outcomes after the trial commenced, with reasons | NA |
| Sample size | 7a | How sample size was determined | 6 |
|  | 7b | When applicable, explanation of any interim analyses and stopping guidelines | 20 |
| Randomisation: |  |  |  |
| Sequence generation | 8a | Method used to generate the random allocation sequence | 5 |
|  | 8b | Type of randomisation; details of any restriction (such as blocking and block size) | 5 |
| Allocation concealment mechanism | 9 | Mechanism used to implement the random allocation sequence (such as sequentially numbered containers), describing any steps taken to conceal the sequence until interventions were assigned | 5 |
| Implementation | 10 | Who generated the random allocation sequence, who enrolled participants, and who assigned participants to interventions | 5 |
| Blinding | 11a | If done, who was blinded after assignment to interventions (for example, participants, care providers, those assessing outcomes) and how | 8 |
|  | 11b | If relevant, description of the similarity of interventions |  |
| Statistical methods | 12a | Statistical methods used to compare groups for primary and secondary outcomes | 6-8 |
|  | 12b | Methods for additional analyses, such as subgroup analyses and adjusted analyses | 6-8 |
| Results | | | |
| Participant flow (a diagram is strongly recommended) | 13a | For each group, the numbers of participants who were randomly assigned, received intended treatment, and were analysed for the primary outcome | results chapters |
|  | 13b | For each group, losses and exclusions after randomisation, together with reasons | results chapters |
| Recruitment | 14a | Dates defining the periods of recruitment and follow-up | 7, 20-22 |
|  | 14b | Why the trial ended or was stopped | 7 |
| Baseline data | 15 | A table showing baseline demographic and clinical characteristics for each group | results chapters |
| Numbers analysed | 16 | For each group, number of participants (denominator) included in each analysis and whether the analysis was by original assigned groups | 6-8 and results chapters |
| Outcomes and estimation | 17a | For each primary and secondary outcome, results for each group, and the estimated effect size and its precision (such as 95% confidence interval) | results chapters |
|  | 17b | For binary outcomes, presentation of both absolute and relative effect sizes is recommended | results chapters |
| Ancillary analyses | 18 | Results of any other analyses performed, including subgroup analyses and adjusted analyses, distinguishing pre-specified from exploratory | results chapters |
| Harms | 19 | All important harms or unintended effects in each group (for specific guidance see CONSORT for harms) | results chapters |
| Discussion | | | |
| Limitations | 20 | Trial limitations, addressing sources of potential bias, imprecision, and, if relevant, multiplicity of analyses | results chapters |
| Generalisability | 21 | Generalisability (external validity, applicability) of the trial findings | results chapters |
| Interpretation | 22 | Interpretation consistent with results, balancing benefits and harms, and considering other relevant evidence | results chapters |
| Other information | | |  |
| Registration | 23 | Registration number and name of trial registry | viii |
| Protocol | 24 | Where the full trial protocol can be accessed, if available | Appendix 1 |
| Funding | 25 | Sources of funding and other support (such as supply of drugs), role of funders | 20 + 111 |

**Figure S3.** CONSORT diagram for patella comparison. Reproduced from Figure 3 in Murray et al 2014 [2].

|  |  | Participants randomised  1715 | | | | |
| --- | --- | --- | --- | --- | --- | --- |
|  |  |  |  |  |  |  |
|  |  |  |  |  |  |  |
|  |  | Allocated to patella resurfacing | |  | Allocated to no patella resurfacing | |
|  |  | (n=861) | |  | (n=854) | |
| **Baseline status** |  |  |  |  |  |  |
|  |  |  |  |  |  |  |
| Response |  |  | 813 |  |  | 813 |
| Non-response |  |  | 48 |  |  | 41 |
|  |  |  |  |  |  |  |
| **Treatment received** |  |  |  |  |  |  |
| Received allocated intervention |  |  | 696 |  |  | 724 |
| Did not receive allocated intervention |  |  | 165 |  |  | 130 |
| Reasons: |  |  |  |  |  |  |
| Death before surgery |  |  | 1 |  |  | 1 |
| Withdrawn from surgery |  |  | 19 |  |  | 23 |
| Crossover to other KAT intervention |  |  | 129 |  |  | 93 |
| Received uni-compartmental knee |  |  | 3 |  |  | 5 |
| Unclear |  |  | 13 |  |  | 8 |
|  |  |  |  |  |  |  |
| **Ten-year follow-up status** |  |  |  |  |  |  |
| Response |  |  | 459 |  |  | 432 |
| Deceased |  |  | 229 |  |  | 228 |
| Non-response |  |  | 79 |  |  | 84 |
| Declined further follow-up |  |  | 59 |  |  | 69 |
| Lost to follow-up |  |  | 4 |  |  | 4 |
| Not yet reached ten years |  |  | 11 |  |  | 13 |
| Death before surgery |  |  | 1 |  |  | 1 |
| Withdrawn before surgery |  |  | 19 |  |  | 23 |
|  |  |  |  |  |  |  |
| **Included in primary outcome analysis** |  |  |  |  |  |  |
| Yes |  |  | 816 |  |  | 798 |
| No |  |  | 45 |  |  | 56 |
| Reasons: |  |  |  |  |  |  |
| Death before surgery |  |  | 1 |  |  | 1 |
| Withdrawn before surgery |  |  | 19 |  |  | 23 |
| Death before three-month follow-up |  |  | 6 |  |  | 9 |
| No post-surgery primary outcome |  |  | 19 |  |  | 23 |
|  |  |  |  |  |  |  |
| **Included in economic evaluation** |  |  |  |  |  |  |
| Yes |  |  | 841 |  |  | 830 |
| No |  |  | 20 |  |  | 24 |
| Reasons: |  |  |  |  |  |  |
| Death before surgery |  |  | 1 |  |  | 1 |
| Withdrawn before surgery |  |  | 19 |  |  | 23 |

Figure S4. CONSORT diagram mobile versus fixed bearing. Reproduced from Figure 23 in Murray et al 2014 [2].

|  |  | Participants randomised  539 | | | | |
| --- | --- | --- | --- | --- | --- | --- |
|  |  |  |  |  |  |  |
|  |  |  |  |  |  |  |
|  |  | Allocated to mobile bearing | |  | Allocated to no fixed bearing | |
|  |  | (n=276) | |  | (n=263) | |
| **Baseline status** |  |  |  |  |  |  |
|  |  |  |  |  |  |  |
| Response |  |  | 264 |  |  | 252 |
| Non-response |  |  | 12 |  |  | 11 |
|  |  |  |  |  |  |  |
| **Treatment received** |  |  |  |  |  |  |
| Received allocated intervention |  |  | 226 |  |  | 243 |
| Did not receive allocated intervention |  |  | 50 |  |  | 20 |
| Reasons: |  |  |  |  |  |  |
| Death before surgery |  |  | 0 |  |  | 0 |
| Withdrawn from surgery |  |  | 14 |  |  | 8 |
| Crossover to other KAT intervention |  |  | 32 |  |  | 10 |
| Received uni-compartmental knee |  |  | 2 |  |  | 2 |
| Unclear |  |  | 2 |  |  | 0 |
|  |  |  |  |  |  |  |
| **Ten-year follow-up status** |  |  |  |  |  |  |
| Response |  |  | 102 |  |  | 124 |
| Deceased |  |  | 70 |  |  | 55 |
| Non-response |  |  | 25 |  |  | 21 |
| Declined further follow-up |  |  | 16 |  |  | 16 |
| Lost to follow-up |  |  | 0 |  |  | 0 |
| Not yet reached ten years |  |  | 49 |  |  | 38 |
| Death before surgery |  |  | 0 |  |  | 0 |
| Withdrawn before surgery |  |  | 14 |  |  | 8 |
|  |  |  |  |  |  |  |
| **Included in primary outcome analysis** |  |  |  |  |  |  |
| Yes |  |  | 250 |  |  | 249 |
| No |  |  | 22 |  |  | 14 |
| Reasons: |  |  |  |  |  |  |
| Death before surgery |  |  | 0 |  |  | 0 |
| Withdrawn before surgery |  |  | 14 |  |  | 8 |
| Death before three-month follow-up |  |  | 2 |  |  | 1 |
| No post-surgery primary outcome |  |  | 10 |  |  | 5 |
|  |  |  |  |  |  |  |
| **Included in economic evaluation** |  |  |  |  |  |  |
| Yes |  |  | 262 |  |  | 255 |
| No |  |  | 14 |  |  | 8 |
| Reasons: |  |  |  |  |  |  |
| Death before surgery |  |  | 0 |  |  | 0 |
| Withdrawn before surgery |  |  | 14 |  |  | 8 |

Figure S5. CONSORT diagram all–polyethylene versus metal-backed. Reproduced from Figure 39 in Murray et al 2014 [2].

|  |  | Participants randomised  409 | | | | |
| --- | --- | --- | --- | --- | --- | --- |
|  |  |  |  |  |  |  |
|  |  |  |  |  |  |  |
|  |  | Allocated to all-polyethylene | |  | Allocated to metal-backing | |
|  |  | (n=207) | |  | (n=202) | |
| **Baseline status** |  |  |  |  |  |  |
| Response |  |  | 201 |  |  | 198 |
| Non-response |  |  | 6 |  |  | 4 |
|  |  |  |  |  |  |  |
| **Treatment received** |  |  |  |  |  |  |
| Received allocated intervention |  |  | 170 |  |  | 195 |
| Did not receive allocated intervention |  |  | 37 |  |  | 7 |
| Reasons: |  |  |  |  |  |  |
| Death before surgery |  |  | 0 |  |  | 0 |
| Withdrawn from surgery |  |  | 4 |  |  | 3 |
| Crossover to other KAT intervention |  |  | 31 |  |  | 1 |
| Received uni-compartmental knee |  |  | 1 |  |  | 1 |
| Unclear |  |  | 1 |  |  | 2 |
|  |  |  |  |  |  |  |
| **Ten-year follow-up status** |  |  |  |  |  |  |
| Response |  |  | 85 |  |  | 91 |
| Deceased |  |  | 58 |  |  | 55 |
| Non-response |  |  | 19 |  |  | 16 |
| Declined further follow-up |  |  | 18 |  |  | 16 |
| Lost to follow-up |  |  | 0 |  |  | 0 |
| Not yet reached ten years |  |  | 23 |  |  | 51 |
| Death before surgery |  |  | 0 |  |  | 0 |
| Withdrawn before surgery |  |  | 4 |  |  | 3 |
|  |  |  |  |  |  |  |
| **Included in primary outcome analysis** |  |  |  |  |  |  |
| Yes |  |  | 196 |  |  | 192 |
| No |  |  | 11 |  |  | 10 |
| Reasons: |  |  |  |  |  |  |
| Death before surgery |  |  | 0 |  |  | 0 |
| Withdrawn before surgery |  |  | 4 |  |  | 8 |
| Death before three-month follow-up |  |  | 1 |  |  | 1 |
| No post-surgery primary outcome |  |  | 6 |  |  | 5 |
|  |  |  |  |  |  |  |
| **Included in economic evaluation** |  |  |  |  |  |  |
| Yes |  |  | 203 |  |  | 199 |
| No |  |  | 4 |  |  | 3 |
| Reasons: |  |  |  |  |  |  |
| Death before surgery |  |  | 0 |  |  | 0 |
| Withdrawn before surgery |  |  | 4 |  |  | 3 |

References

1. Schulz KF, Altman DG, Moher D. CONSORT 2010 Statement: updated guidelines for reporting parallel group randomised trials. BMC Med. 2010;8:18. doi:1741-7015-8-18 [pii] 10.1186/1741-7015-8-18.

2. Murray DW, MacLennan GS, Breeman S, Dakin HA, Johnston L, Campbell MK et al. A randomised controlled trial of the clinical effectiveness and cost-effectiveness of different knee prostheses: the Knee Arthroplasty Trial (KAT). Health Technology Assessment. 2014;18. doi:10.3310/hta18190.
